# Overcoming resistance to immune checkpoint inhibitor in non-small cell lung cancer: the promise of combination therapy

**DOI:** 10.3389/fimmu.2025.1691980

**Published:** 2025-11-05

**Authors:** Xuebing Shi, Wenxia Deng, Yunlei Pan, Yingchun Chen, Yinke Wang, Jing Wu

**Affiliations:** ^1^ Thoracic Oncology Ward of Cancer Center, Tongling People’s Hospital, Tongling, Anhui, China; ^2^ Department of Nuclear Medicine, Tongling People’s Hospital, Tongling, Anhui, China; ^3^ Research Department of Tongling People’s Hospital, Tongling, Anhui, China

**Keywords:** NSCLC, ICI, immune resistance, tumor microenvironment, combination therapy

## Abstract

The clinical application of immune checkpoint inhibitor (ICI) has profoundly reshaped the therapeutic landscape of non-small cell lung cancer (NSCLC), heralding a new era of immunotherapy in oncology. However, despite the durable and remarkable clinical benefits observed in a subset of patients, a considerable proportion exhibit primary or acquired resistance, substantially limiting overall therapeutic efficacy. Immune resistance has emerged as one of the central challenges in ICI-based NSCLC treatment, stemming from an incomplete understanding of ICI mechanisms of action and the highly heterogeneous and dynamically complex nature of the NSCLC tumor microenvironment (TME). This review provides a comprehensive overview of the diverse molecular and cellular mechanisms underlying ICI resistance in NSCLC, highlights recent advances in combination therapeutic strategies aimed at overcoming resistance, and discusses the opportunities and challenges associated with their clinical translation and application.

## 1 Introduction

According to the 2025 cancer statistics, lung cancer ranks second in incidence and first in mortality among all cancer types (1). Pathologically, lung cancer is broadly classified into two major types: non-small cell lung cancer (NSCLC) and small cell lung cancer (SCLC), with NSCLC accounting for approximately 85% of all cases, making it the most common subtype. NSCLC can be further subdivided into squamous cell carcinoma, adenocarcinoma, and large cell carcinoma. Currently, for patients with resectable or locally advanced NSCLC, surgical resection followed by adjuvant chemotherapy remains the standard of care. In contrast, for those with locally advanced unresectable NSCLC, definitive concurrent chemoradiotherapy followed by consolidation immunotherapy or targeted therapy is generally recommended according to current clinical guidelines (2–4). However, the prognosis of NSCLC remains poor, with a substantial proportion of patients failing to achieve a 5-year survival even after curative-intent treatment (5).

Over the past decade, rapid advances in genomics and immunotherapy have revolutionized the therapeutic landscape of NSCLC (6). Immune checkpoint inhibitor (ICI), represented by anti- programmed cell death protein 1(PD-1)/programmed cell death ligand 1(PD-L1) and anti-cytotoxic T lymphocyte-associated antigen-4(CTLA-4) antibodies, have markedly improved clinical outcomes and extended survival in selected patient populations. Multiple landmark clinical trials have demonstrated the robust efficacy of ICI in NSCLC (7–9), ushering in a new era of cancer immunotherapy.

Despite the promising prospects that immunotherapy has brought to patients with NSCLC, only a subset of individual derives durable clinical benefit (10). A major challenge in current clinical practice is that the majority of patients eventually develop resistance to ICI after treatment. Multiple clinical trials have shown that more than 60% of treatment-naïve NSCLC patients fail to respond to ICI therapy (11–13). Although the addition of chemotherapy to immunotherapy can achieve transient tumor control in some patients, most of these individuals ultimately develop acquired resistance after an initial period of benefit. This phenomenon underscores the fact that our current understanding of the mechanisms underlying immunotherapy resistance remains incomplete. Future studies are needed to elucidate the complex biological processes driving ICI resistance, thereby providing a rational basis for overcoming therapeutic resistance in NSCLC.

## 2 Definition and characteristics of immune resistance

The concepts of primary and acquired resistance were originally derived from the context of chemotherapeutic anticancer strategies. However, in the field of immunotherapy, there is still no unified consensus regarding resistance patterns. Currently, one of the more authoritative definitions of immunotherapy resistance is proposed by the Society for Immunotherapy of Cancer, which classifies resistance to ICI therapy into three categories: primary resistance, acquired resistance, and disease progression occurring after treatment discontinuation for any reason (this review will not conduct an in-depth analysis of this point) (14).

**Figure 1:**
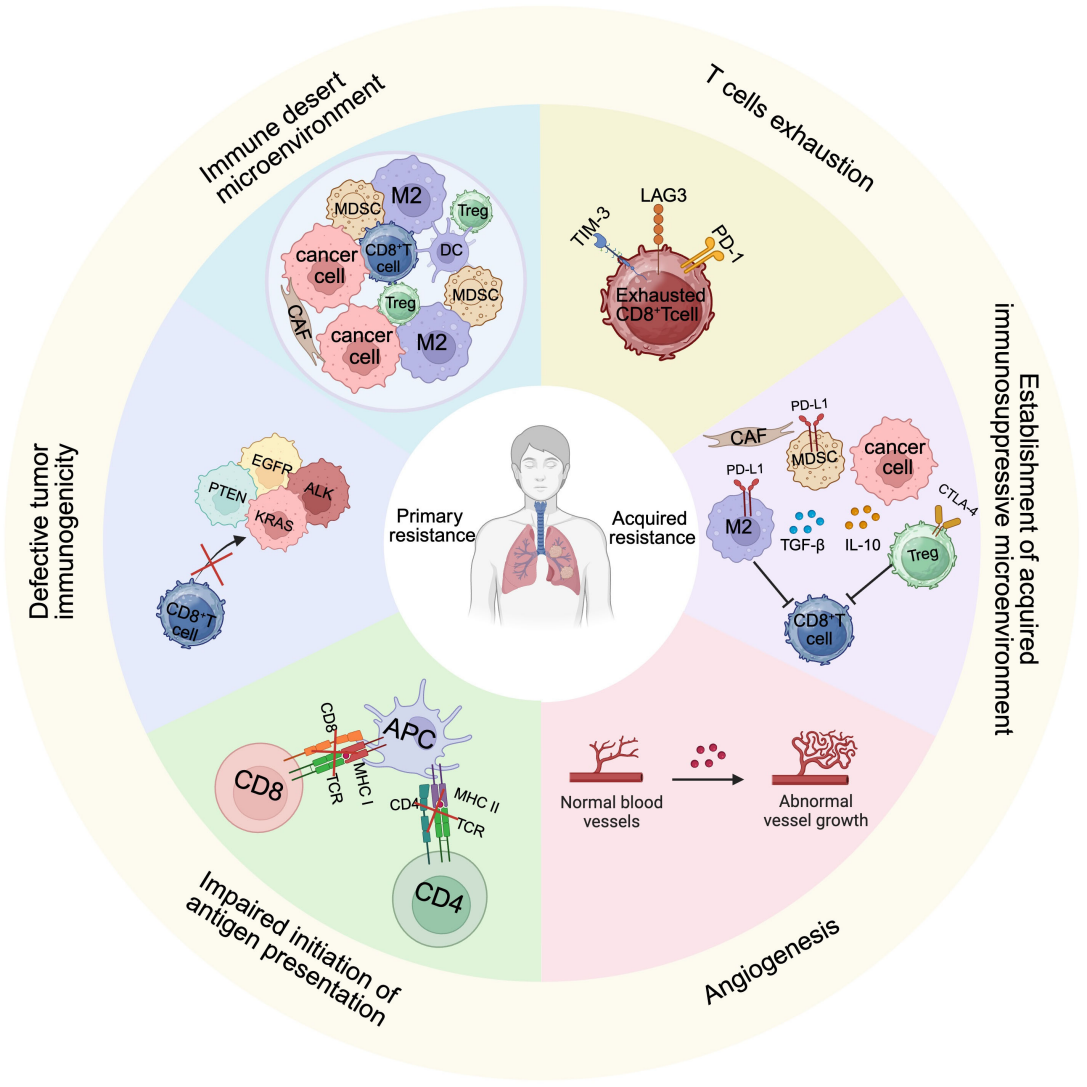
Differences in characteristics of primary and secondary resistance to ICI immunotherapy. Primary resistance is characterized by the innate formation of an immune desert TME, antigen presentation defects leading to failure of adaptive immune activation, and partial gene mutations in tumor cells leading to strong drug resistance. The hallmark of secondary resistance lies in the presence of initially activated T cells within the TME. However, with persistent antigenic stimulation, these T cells gradually undergo functional exhaustion, leading to the establishment of an immunosuppressive TME.

Primary resistance refers to the inability of patients to derive clinical benefit from initial immunotherapy, characterized by the absence of objective response, continued disease progression, or rapid deterioration. More specifically, it is defined as tumor response or prolonged stable disease (SD, according to RECIST version 1.1) lasting less than six months, although the exact time threshold may vary depending on tumor type. This form of resistance substantially limits the broad applicability of immunotherapy and represents a major bottleneck in the advancement of precision cancer immunotherapy. Mechanistically, primary resistance arises from tumor immune escape programs that are already established prior to the initiation of immunotherapy. It is typically associated with profound CD8^+^T cells exhaustion, high infiltration of immunosuppressive cell populations, and intrinsic defects in tumor immunogenicity that impair the activation of effective antitumor immunity (15). Secondary resistance, also referred to as acquired resistance, is defined as the progression of disease during continued treatment in patients who have previously achieved a documented and confirmed objective response or prolonged stable disease (SD > 6 months). Unlike primary resistance, acquired resistance emerges during therapy as a result of a dynamic “arms race” between the tumor and the immune system, whereby the tumor initially responds well to ICI but subsequently regains proliferative advantages and escapes immune surveillance. Mechanistically, acquired resistance is more complex and can be understood biologically as an adaptive process in which tumor cells undergo genetic and phenotypic changes in response to evolving conditions within the tumor microenvironment (TME) (15, 16). These changes may include the emergence of resistance-associated gene mutations, defects in antigen presentation, and the establishment of an immunosuppressive milieu (17, 18)(**Figure 1**). Based on the density, distribution, and functional state of immune cells, the NSCLC TME can be broadly categorized into four conserved immune phenotypes: immunologically active, immunosuppressed, immune-excluded, and immune-desert subtypes (19, 20). These phenotypes not only depict the spatial and cellular landscape of the TME but also provide mechanistic insight into both primary and acquired ICI resistance.The immunologically active phenotype (also known as “hot tumors”) is characterized by abundant infiltration of functional CD8^+^ cytotoxic T cells and other effector lymphocytes, activation of IFN-γ signaling, and upregulation of PD-L1 expression (21). NSCLC patients with this phenotype—often defined by high PD-L1 tumor proportion score—tend to respond favorably to ICI (22, 23). However, even within this group, acquired resistance can emerge through progressive T cell exhaustion, antigen loss, or adaptive upregulation of alternate immune checkpoints. Thus, the immunologically active subtype represents both the ideal responder and the evolutionary battlefield where dynamic immune escape develops during therapy.

The immunosuppressed phenotype is characterized by the presence of immune infiltrates that are rendered dysfunctional by dominant suppressive mechanisms—such as regulatory T cells (Tregs), myeloid-derived suppressor cells (MDSCs), or M2-like tumor-associated macrophages (24, 25). Therefore, patients with this subtype of NSCLC have activated immune systems, but due to the presence of a large number of immunosuppressive cell populations, patients are very likely to develop acquired resistance after receiving ICI immunotherapy.

In contrast, the immune-excluded phenotype shows abundant T cells that accumulate around the tumor stroma or invasive margin but fail to infiltrate the tumor parenchyma (26). This spatial immune barrier is maintained by cancer-associated fibroblasts (CAFs), dense extracellular matrix (ECM) deposition, and TGF-β signaling activation (27–29). Immune-excluded tumors often exhibit innate primary resistance to ICIs because the immune system remains physically separated from tumor cells. Strategies disrupting CAF-TGF-β signaling or ECM remodeling have been proposed to convert this phenotype into a more inflamed, ICI-responsive state (30).

the immune-desert phenotype represents the prototypical “cold tumor, “ defined by an absence of lymphocyte infiltration and deficient antigen presentation or T-cell priming (31, 32). These tumors are almost universally refractory to ICI monotherapy (33), as no pre-existing immune activation exists to be re-invigorated. Here, primary resistance is driven by the failure of immune initiation rather than suppression. Therapeutic strategies aimed at converting “cold” tumors into “hot” ones—such as oncolytic viruses, tumor vaccines, or innate immune agonists—are being explored to overcome this intrinsic resistance (34, 35).

## 3 Mechanisms of immune resistance in NSCLC

**Figure 2:**
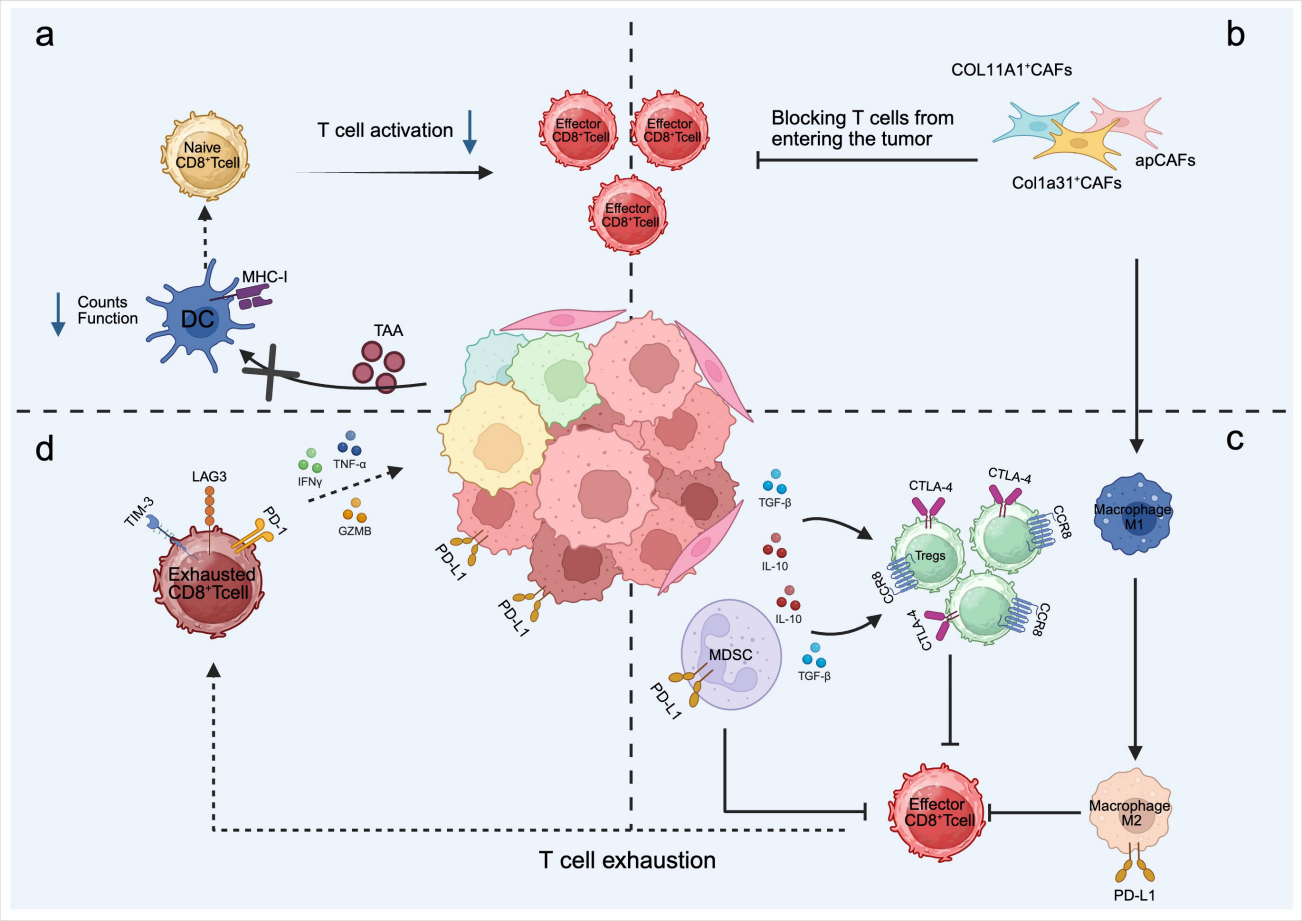
Mechanism of ICI resistance in NSCLC. **(a)** Tumor cells gene mutations or immunosuppression lead to a decrease in the counts of dendritic cells (DCs) in the TME and defective antigen presentation function, which in turn leads to the inability of T cells to fully activate and differentiate into effector T cells. **(b)** CAFs act as a barrier around the tumor, limiting the infiltration of effector CD8^+^T cells into the tumor parenchyma, thereby inhibiting their anti-tumor function. additionally, CAFs can induce Tregs proliferation and polarization of M1 macrophages to M2, establishing a tumor suppressive immune microenvironment. **(c)** The immunosuppressive tumor microenvironment harbors abundant immunosuppressive cell populations, including MDSCs, Tregs, M2 macrophages, as well as various inhibitory cytokines, which collectively impose profound constraints on the antitumor activity of CD8^+^ T cells and accelerate their exhaustion and dysfunctional differentiation. **(d)** Chronic antigen stimulation drives CD8^+^ T cells into a terminally exhausted state, characterized by the upregulation of inhibitory receptors such as PD-1, TIM-3, and LAG-3 on their surface, along with diminished effector cytokine secretion capacity, ultimately resulting in the loss of antitumor functionality.

While ICI have achieved breakthroughs in the treatment of NSCLC, their clinical efficacy is limited by widespread primary and secondary resistance. ICI resistance is not driven by a single factor but rather by a multi-layered, multi-step process encompassing abnormalities in tumor cell-intrinsic genes and signaling pathways, impaired antigen presentation and recognition, and immunosuppressive cell populations within the TME. Systematically elucidating these mechanisms is crucial for uncovering the fundamental mechanisms of tumor immune escape and laying the theoretical and practical foundation for optimizing immunotherapy strategies, developing novel combination therapies, and exploring potential therapeutic targets (**Figure 2**).

### 3.1 CD8^+^ T cells exhaustion

Accumulating evidence indicates that CD8^+^ T cells exhaustion is one of the key mechanisms underlying tumor resistance to ICI therapy. Lung cancer progression has been closely linked to CD8^+^ T cells dysfunction. In the context of acquired resistance to immunotherapy, CD8^+^ T cells in lung cancer often display elevated expression of multiple inhibitory receptors, such as PD-1, T cell immunoglobulin and mucin-domain containing-3(TIM-3), and Lymphocyte activation gene-3(LAG-3) (36, 37). A recent large-scale study in patients with NSCLC further demonstrated that the co-expression of exhaustion markers on CD8^+^T cells—including PD-1, T cell immunoreceptor with Ig and ITIM domains(TIGIT), LAG-3, and TIM-3—serves as an independent predictive factor for the occurrence of primary resistance to ICI (38). Moreover, increased serum levels of soluble TIM-3 (sTIM-3) have been detected in NSCLC patients who fail to respond to αPD-1 therapy, suggesting its potential role as a biomarker of primary immune resistance and its capacity to mediate immunosuppression through mechanisms independent of membrane-bound TIM-3 (39). Notably, sustained Interferon-γ(IFN-γ) stimulation has also been shown to contribute to acquired immune resistance in NSCLC, a process often accompanied by a substantial accumulation of terminally exhausted T cells and Tregs within the TME, further dampening antitumor immune responses (40).

With the rapid advancement of single-cell transcriptomics, researchers have identified progenitor exhausted T cells (Tpex) as key mediators of response to ICI therapy. This subset, characterized by the co-expression of PD-1 and TCF-1, can rapidly expand during αPD-1/PD-L1 treatment and differentiate into highly functional effector T cells. Analyses of paired tumor biopsy samples before and after ICI therapy have shown that patients with a low abundance of Tpex cells are more likely to exhibit non-responsiveness to αPD-1 treatment (41). However, even under PD-1/PD-L1 blockade, Tpex cells may fail to undergo the necessary epigenetic reprogramming required for effective differentiation into terminally exhausted CD8^+^T cells (Tex-term), thereby contributing to immunotherapy resistance (42). Notably, the presence of Tpex cells has been strongly correlated with longer progression-free survival (PFS) and greater clinical benefit in patients with NSCLC receiving ICI treatment (43). Furthermore, functional impairment of tumor antigen-specific CD8^+^ tissue-resident memory T cells have also been implicated in the development of primary resistance to neoadjuvant PD-1 blockade in NSCLC patients (44).

### 3.2 Tumor immunosuppressive microenvironment

#### 3.2.1 Tregs

Tregs, as a critical subset of CD4^+^T cells, exert profound immunosuppressive effects within the TME, thereby contributing to the establishment of an immunosuppressive niche and representing an important mechanism of resistance to immunotherapy (45, 46). Among them, CCR8^+^Tregs are recognized as a prototypical highly suppressive subset, whose dense infiltration within the TME markedly limits the therapeutic efficacy of αPD-1 antibodies in NSCLC (47). Single-cell atlas analyses of NSCLC patients before and after αPD-1 therapy have revealed that patients enriched with Tregs in the tumor immune microenvironment exhibit a lower major pathological response rate, a phenomenon closely associated with an increased proportion of Tex-term and clonal expansion of CCR8^+^Tregs (48).

In addition, the OX40^hi^ GITR^hi^ Treg subset displays stronger immunosuppressive activity, and its accumulation in the TME has been significantly linked to resistance to αPD-1 therapy (49). PD-1^+^Tregs are also considered a functionally potent suppressive subset, whose enrichment in tumors such as NSCLC is strongly associated with immunotherapy resistance (50). In *Kras* ^G12D/+^; *p53* ^-/-^ NSCLC mouse model, it was further demonstrated that αPD-1 antibodies fail to synergize with anti-angiogenic agents to enhance antitumor effects, with the underlying resistance mechanism closely tied to macrophage-driven accumulation of PD-1^+^Tregs within the TME (51).

#### 3.2.2 Monocytes-macrophages

Tumor-associated macrophages (TAMs) infiltration is closely associated with resistance to ICI therapy and shows no significant correlation with PD-L1 expression levels. Notably, genes associated with M2 polarization, such as *BCL2*, are significantly upregulated in non-responders to ICI (52). Another study demonstrated that in the TME of ICI-nonresponsive NSCLC patients, SPP1^+^macrophages and COL11A1^+^ fibroblasts form highly enriched cellular networks that suppress T cells infiltration into the tumor parenchyma via intercellular interactions (53). In addition, overexpression of the cystine transporter SLC7A11 in TAM has been strongly linked to αPD-L1 resistance. In murine models, macrophage-specific deletion of SLC7A11 markedly attenuates M2 polarization and enhances CD8^+^ T cells recruitment, thereby suppressing lung cancer progression (54). Of note, abundant infiltration of M2-type TAMs has also been implicated in ICI-induced hyperprogressive disease. In pre-treatment surgical specimens from all NSCLC patients who developed HPD, a dense accumulation of CD163^+^CD33^+^PD-L1^+^epithelioid macrophages were observed (55).

#### 3.2.3 MDSCs

Multiple studies have demonstrated that myeloid-derived suppressor cells (MDSCs) play a pivotal role in mediating resistance to ICIs in NSCLC. An exploratory analysis reported that elevated peripheral levels of monocytic MDSCs in NSCLC patients are strongly associated with primary resistance to ICI therapy (56). Further evidence indicates that in NSCLC patients with high PD-L1 expression but poor response to ICIs, the IL-6/JAK/STAT3 signaling pathway is aberrantly activated, with MDSCs identified as the predominant source of IL-6. This finding suggests that MDSCs contribute to immune resistance via cytokine-mediated signaling regulation (57).

In addition, tumor cell–derived CXCL5 has been shown to promote the recruitment of tumor-associated neutrophils into the lung parenchyma, thereby inducing CD8^+^ T cells exhaustion and facilitating ICI resistance (58). A retrospective analysis of NSCLC patients receiving ICI identified four cases of early tumor progression during treatment. These patients not only exhibited poor infiltration of tumor-reactive CD8^+^ T cells but also showed substantial accumulation of MDSCs and M2-type TAMs (59). Moreover, in NSCLC with *LKB1* deficiency, abnormal enrichment of granulocytic MDSCs within the TME has been recognized as a key mechanism underlying resistance to ICI (60).

#### 3.2.4 CAFs

CAFs constitute the major extracellular matrix–producing cell population within the TME of NSCLC. Recent studies have revealed their pronounced heterogeneity and multiple functional subtypes, which play complex and pivotal roles in immune regulation and the development of immune resistance (61–63). Multi-omics analyses have demonstrated that COL11A1^+^ CAFs are enriched at the tumor margins of ICI-nonresponsive NSCLC, where they impede contact between tumor cells and cytotoxic T lymphocytes and cooperate with SPP1^+^macrophages to promote immune resistance (53).In another study, abundant infiltration of antigen-presenting CAFs (apCAFs) within the TME was shown to induce Treg proliferation, thereby establishing a highly suppressive immune-resistant milieu (64). Similarly, FAP^+^αSMA^+^CAFs and MYH11^+^αSMA^+^ CAFs have been implicated in primary resistance to ICIs in mature tertiary lymphoid structure positive NSCLC, contributing to the formation of an immunosuppressive microenvironment (29). Col13a1^+^ CAFs can recruit macrophages and Tregs, impair DCs and CD8^+^ T cells function, and promote pulmonary fibrosis, collectively generating an immunosuppressive TME that drives ICI resistance in NSCLC (65). Moreover, IFN-γ has been reported to stimulate the expansion of apCAFs within the TME, leading to upregulation of programmed cell death ligand 2 (PD-L2) expression. This, in turn, triggers the accumulation of FOXP1^+^Tregs via the PD-L2–RGMB axis, ultimately contributing to non-responsiveness to immunotherapy in NSCLC patients (64).

### 3.3 Antigen presentation defect

Defective antigen presentation has been recognized as a critical mechanism underlying ICI resistance in NSCLC (66, 67). Approximately 40% of NSCLC harbor allele-specific loss of heterozygosity in human leukocyte antigen class I (*HLA-I LOH*), a genomic alteration associated with increased neoantigen burden, PD-L1 positivity, and poor clinical response to ICI (68, 69). β2-microglobulin (*B2M*), an essential molecular chaperone for HLA-I–mediated antigen presentation, is frequently lost in NSCLC, resulting in an immunosuppressive TME characterized by reduced tumor-infiltrating lymphocytes and ICI resistance (67, 70).

DCs, the most potent antigen-presenting cells in the TME, are indispensable for antigen presentation and the priming of CD8^+^ T cells. High DC signature genes are associated with better overall survival (OS) and clinical benefit in NSCLC patients receiving atezolizumab (71). In *KEAP1*-mutant tumors, the accumulation and activation of CD103^+^ DCs are markedly impaired, thereby attenuating CD8^+^ T cells–mediated antitumor immunity (72).

Hypoxia is a defining feature of the TME and can promote epithelial–mesenchymal transition via HIF-1α, thereby enhancing tumor cell migration and invasiveness (73). Transcriptomic analysis of hypoxia signatures across TCGA datasets revealed that lung squamous cell carcinoma (LUSC) is among the most hypoxic tumor types, whereas lung adenocarcinoma (LUAD) exhibits moderate overall hypoxia but the greatest intratumoral heterogeneity in hypoxia levels (74). Recently, a study investigating hypoxia-associated ICI resistance in NSCLC found that tumors with acquired ICI resistance frequently contained extensive hypoxic regions, which coincided with reduced infiltration of CD8^+^ T cells and downregulation of both MHC-I and MHC-II expression on tumor cells (75).

### 3.4 Defective tumor immunogenicity

A spatial TME analysis of Kirsten rat sarcoma viral oncogene homolog (*KRAS*)-mutant NSCLC patients undergoing ICI therapy revealed that infiltration of CD68^+^ macrophages and PanCK^+^/CD33^+^/FOXP3^+^ cells is associated with resistance to ICI (76). Studies have confirmed that NSCLC tumors harboring the *KRAS* ^G12D^ mutation exhibit a more immunosuppressive TME and respond poorly to PD-1/PD-L1 blockade (77). The *KRAS* ^G12D^ mutation mediates αPD-1 resistance by downregulating PD-L1 expression and reducing CD8^+^ T cell infiltration in NSCLC (78).

A phase III-controlled trial (NCT01673867) identified *STK11*/*KEAP1* mutations as the most prevalent genomic drivers of primary resistance to αPD-1/L1 therapy in *KRAS*-mutant lung adenocarcinoma (79). In the phase III CheckMate 057 trial evaluating nivolumab versus docetaxel in previously treated NSCLC, the objective response rate to PD-1 blockade in the *LKB1*-mutant subgroup was only 7.4%, compared with over 35% in the *LKB1* wild-type subgroup (*P* < 0.001). Median OS was 16 months for *LKB1* wild-type patients but decreased significantly to 6.4 months for those harboring *LKB1* mutations (*P* = 0.0045) (80). Furthermore, multiple studies have demonstrated that STK11/KEAP1 mutations in NSCLC contribute to the establishment of an immunosuppressive TME, ultimately resulting in primary resistance to αPD-1 therapy (81–86).

Clinical studies have reported that NSCLC patients harboring epidermal growth factor receptor (*EGFR*) mutations fail to derive clinical benefit from monotherapy with αPD-1/L1 inhibitors, regardless of PD-L1 expression levels (87–89). Moreover, the combination of EGFR tyrosine kinase inhibitors with αPD-1/L1 therapy has not yielded significant clinical improvements and is associated with increased treatment-related toxicity (90). Consequently, current guidelines do not recommend combining tyrosine kinase inhibitors with ICI for *EGFR*-mutant NSCLC patients, underscoring the urgent need to develop alternative immunotherapeutic approaches, such as CAR-T cell therapy, for this subgroup.

Comparative analyses of immune characteristics before and after the development of acquired resistance to ICI in NSCLC patients have identified various gene mutations including *B2M*, *STK11*, *KEAP1*, and *JAK1/2* associated with resistance (91). Additionally, multiple studies have reported *PTEN* mutations in NSCLC patients unresponsive to ICI (92, 93). Preclinical models have demonstrated that *PTEN* loss is a critical driver of αPD-1 resistance in NSCLC, primarily through increased infiltration of Tregs in the TME—a phenomenon also observed in patients (94).

### 3.5 Histological transformation to SCLC

Transformation of NSCLC to SCLC following immunotherapy is considered a distinct mechanism of immune resistance in lung cancer. Multiple case reports have documented histological transformation from NSCLC to SCLC in patients who developed resistance to ICI (95–98). Accumulating evidence supports a close association between such histologic transformation and immunotherapy resistance. However, the underlying mechanisms remain incompletely understood. Current research proposes two possible models: the mixed‐component selection hypothesis and the common progenitor cell transformation hypothesis (99). The mixed‐component selection hypothesis suggests that both NSCLC and SCLC cell populations coexist within the TME. Under immunotherapy, NSCLC cells—being more sensitive—are eliminated, whereas SCLC cells, inherently resistant, undergo clonal expansion. This hypothesis, however, still lacks validation from large-scale clinical cohorts. In contrast, the common progenitor cell transformation hypothesis posits that NSCLC and SCLC originate from shared tumor stem cells, and under therapeutic pressure, NSCLC cells may transdifferentiate into SCLC. Several reports have shown that transformed SCLC retains the original driver mutations of NSCLC while acquiring additional genetic alterations that ultimately lead to histologic conversion (95, 97, 100, 101). Although current evidence more strongly favors the common progenitor cell model, the precise molecular and cellular mechanisms underlying this process warrant further investigation.

## 4 Combination therapy strategies to reverse ICI resistance

**Figure 3:**
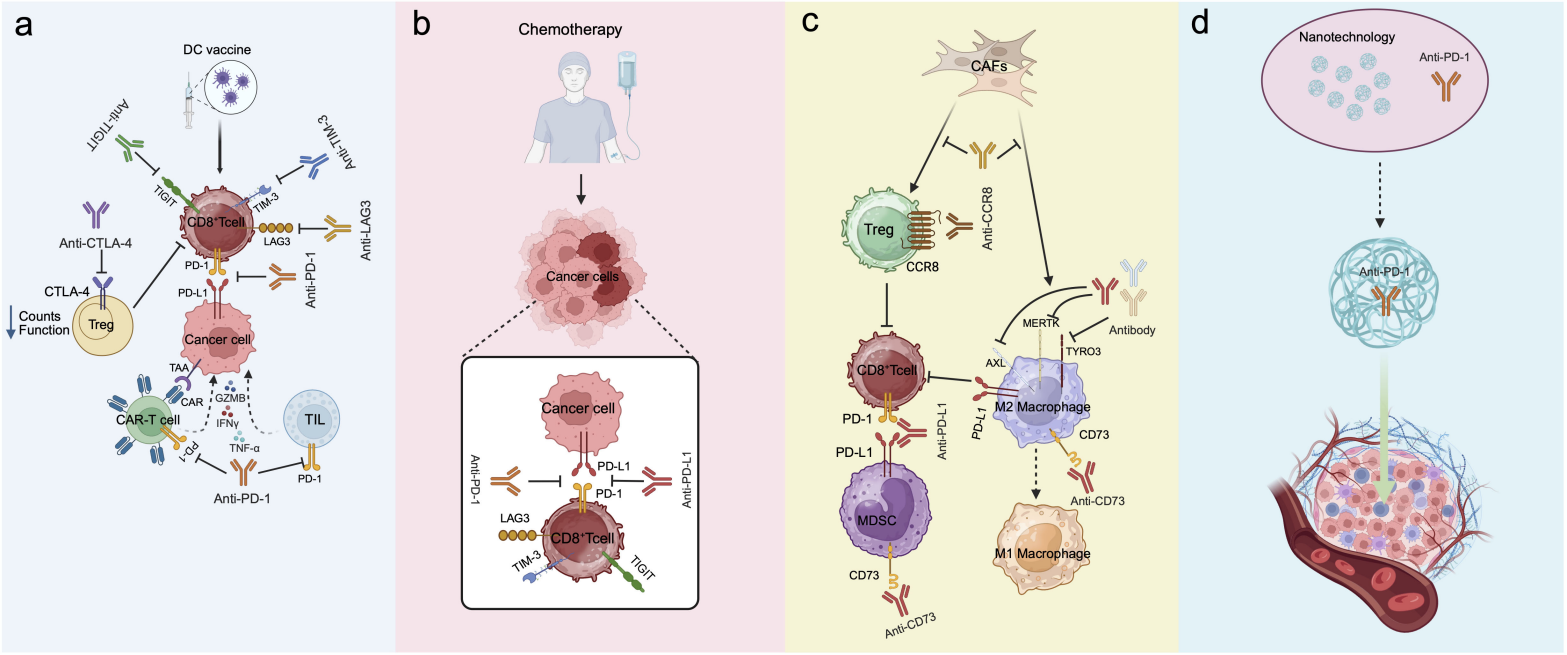
Combination therapy strategy to reverse ICI immune resistance. **(a)** Dual ICI combination therapy reverses CD8^+^T cells exhaustion. Meanwhile, αPD-1/L1 combined immunotherapy activates the adaptive immune response of CD8^+^T cells and enhances their anti-tumor function. **(b)** ICI combined with chemotherapy induce immunogenic tumor cell death, release tumor associated antigens and activate CD8^+^T cells to enhance antitumor immunity. **(c)** Targeting immunosuppressive cells or cytokines in the TME promotes the formation of a “hot tumor” microenvironment, and in combination with ICI, restores CD8^+^T cells anti-tumor function. **(d)** Nanotechnology encapsulates PD-1/L1 antibodies, enabling their delivery to the tumor parenchyma, further amplifying the anti-tumor effect of PD-1/L1 antibodies.

In recent years, with the deepening understanding of the mechanisms of resistance to ICI, combination therapy has become an important approach to overcoming this resistance barrier. Combining ICI with chemoradiotherapy, immunotherapy, targeted inhibition, and nanomaterials can improve the tumor immune microenvironment in multiple dimensions, enhancing antigen presentation and T cells effector function, thereby synergistically improving therapeutic efficacy. Systematically exploring the advantages and challenges of different combination modalities is crucial for optimizing treatment regimens, prolonging patient benefits, and promoting precision immunotherapy (**Figure 3**).

### 4.1 Dual ICIs combination therapy

Resistance to PD-1 blockade is frequently accompanied by the compensatory upregulation of alternative inhibitory receptors such as CTLA-4, TIGIT, TIM-3, or LAG-3, which sustain T cell dysfunction and maintain an immunosuppressive tumor milieu. Dual ICI therapy aims to interrupt these parallel suppressive pathways, thereby restoring durable antitumor immunity. Such combinations not only potentiate the initial immune activation but may also delay or reverse adaptive resistance driven by checkpoint redundancy.

#### 4.1.1 αPD-1/L1 combined with αCTLA-4

Results from multiple clinical trials (NCT01454102 and NCT02477826) have shown that nivolumab plus ipilimumab combination therapy provides superior clinical benefits to nivolumab monotherapy in NSCLC patients (102, 103). However, the situation is more complicated in NSCLC patients who are resistant to αPD-1/L1 therapy. The phase II S1400F trial (NCT03373760) found that durvalumab (αPD-L1) plus tremelimumab(αCTLA-4) failed to produce meaningful clinical responses in squamous NSCLC patients who had developed resistance to prior αPD-1 monotherapy (104). The AK104–202 study (NCT04172454) found that cadonilimab (a bispecific antibody targeting PD-1 and CTLA-4) did not significantly improve OS in ICI-resistant NSCLC patients; however, OS was markedly longer in the acquired resistance group (13.1 months) compared with the primary resistance group (4.9 months), underscoring the importance of distinguishing primary from acquired resistance in future research (105). Fortunately, recent evidence from the phase IIIb TRITON clinical trial (NCT06008093) further suggests that *STK11* and/or *KEAP1* mutations are associated with resistance to pembrolizumab. Importantly, patients harboring these mutations derived greater benefit from durvalumab plus tremelimumab combined with chemotherapy, whereas durvalumab monotherapy provided no significant clinical advantage (106). Preclinical data also support the rationale for multi-target blockade. The trispecific antibody HC010, which simultaneously targets PD-1, CTLA-4, and VEGF, demonstrated potent antitumor efficacy in models of αPD-1–resistant NSCLC (107). However, results from the NCT03091491 trial demonstrated that patients with *EGFR*-mutant NSCLC exhibited limited therapeutic benefit from either nivolumab monotherapy or the combination of nivolumab and ipilimumab following acquired resistance to EGFR-TKI treatment (13). These findings suggest that EGFR-mutant NSCLC harbors primary resistance to ICI therapy. Together, these findings indicate that dual PD-1 and CTLA-4 blockade holds promise for overcoming ICI resistance in NSCLC, while underscoring the need to differentiate primary from acquired resistance and to develop mutation-specific combination strategies.

#### 4.1.2 αPD-1/L1 combined with αTIGIT

Several clinical trials (NCT05226598, NCT05298423, NCT03563716) are underway to evaluate the combination of vibostolimab and pembrolizumab in patients with NSCLC who have not received any prior treatment. A phase 2 clinical trial (NCT04262856) demonstrated that, in chemotherapy-naïve, PD-L1–positive patients with recurrent or metastatic NSCLC, combination therapy with an αTIGIT antibody and an αPD-1/PD-L1 agent significantly improved overall response rate (ORR) and PFS compared with αPD-1/PD-L1 monotherapy (108). Meanwhile, in the phase 1 MK-7684–001 trial (NCT02964013), vibostolimab administered alone or in combination with pembrolizumab showed favorable safety and modest activity (ORR 5%–7%) in αPD-1/PD-L1–refractory NSCLC, while also exhibiting encouraging antitumor activity (109). However, the phase 2 KeyVibe-002 study (NCT04725188), which evaluated the efficacy and safety of pembrolizumab/vibostolimab in patients with metastatic NSCLC who had progressed after prior immunotherapy and platinum-based doublet chemotherapy, reported that neither MK-7684A monotherapy nor MK-7684A plus docetaxel demonstrated a statistically significant improvement in PFS compared with docetaxel alone (110). Therefore, further studies are needed to confirm the effect of αTIGIT combined with αPD-1/L1 in the treatment of ICI-resistant NSCLC. (**Table 1**).

**Table 1:** Clinical trials of αPD-1/L1 combined with other ICI treatments in ICI-resistant NSCLC.

NCT identifier	Eligible patient	Treatment strategy	Phase	Status	Result
Combined with CTLA-4					
NCT03373760	Advanced NSCLC progressing on prior αPD-1/L1 therapy	durvalumab&tremelimumab	II	Completed	Minimal antitumor activity
NCT06008093	Metastatic NSCLC with STK11, KEAP1, or KRAS genetic mutations, incluing NSCLC progressing on prior αPD-1/L1 therapy	durvalumab&tremelimumab	IIIb	Recruiting	\
NCT04172454	Metastatic NSCLC progressing on prior αPD-1/L1 therapy	cadonilimab	Ib/II	Completed	limited efficacy in patients with primary resistance
Combined with TIGIT					
NCT02964013	Anti-PD-1/PD-L1–refractory advanced NSCLC	Vibostolimab& pembrolizumab	I	Completed	Good tolerability and clinical efficacy
Combined with TIM-3					
NCT04931654	Advanced NSCLC progressing on prior αPD-1/L1 therapy	AZD7789	I/IIa	Active, not recruiting	\
Combined with LAG-3					
NCT03625323	PD-X naïve/PD-X refractory NSCLC	IMP321& Pembrolizumab	II	Completed	Good tolerability and clinical efficacy
NCT05785767	NSCLC treated with other ICIs for more than 12 months	Fianlimab& Cemiplimab	2/3	Recruiting	\
NCT05800015	NSCLC treated with other ICIs for more than 12 months	Fianlimab& Cemiplimab	2/3	Recruiting	\
NCT04140500	Advanced NSCLC treated with αPD-1/L1 therapy previously	RO7247669	I	Active, not recruiting	\
NCT05978401	Advanced NSCLC who has previously received ICI (excluding aLAG-3) and have not experienced immunotherapy-related toxicity	GLS-012&zimberelimab	I/II	Not yet recruiting	\

This table lists only the characteristics of the eligible patients related to ICI resistance. Other characteristics of the eligible patients in the cohort can be found on ClinicalTrials.gov.

#### 4.1.3 αPD-1/L1 combined with αTIM-3

Preclinical animal models have revealed that the epithelial/imDC2 axis suppresses the antitumor activity of CD8^+^ tissue-resident memory T cells via the Gal-9/TIM-3 pathway, and that dual targeting of TIM-3 and PD-1 can effectively overcome primary resistance mediated by this axis (111). A recently developed TIM-3/PD-1 bispecific antibody, lomvastomig, has demonstrated superior antitumor activity compared with monotherapy in treating αPD-1–resistant NSCLC, and is currently being evaluated in a phase 1 clinical trial in NSCLC patients (112). In addition, an ongoing phase I/IIa open-label, dose-escalation and expansion study (NCT04931654) is assessing the efficacy of the TIM-3/PD-1 bispecific antibody AZD7789 in NSCLC patients with acquired resistance to αPD-1/PD-L1 therapy. Furthermore, a phase Ib/II, multicenter, open-label trial (NCT06162572) is underway to evaluate the efficacy of the combination therapy of cemiplimab with S095018 (αTIM-3) in treatment-naïve advanced NSCLC patients with high PD-L1 expression.

#### 4.1.4 αPD-1/L1 combined with αLAG-3

A multicenter cohort analysis involving 179 NSCLC patients treated with ICI revealed that high LAG-3 expression on T cells was significantly associated with poor prognosis following ICI therapy (113). The phase II TACTI-002 trial (NCT03625323) evaluated eftilagimod alpha (LAG-3 agonist) in combination with pembrolizumab in two cohorts of NSCLC patients—PD-X naïve (no prior systemic therapy) and PD-X refractory (progressed after prior PD-1/L1 therapy). The study demonstrated encouraging antitumor activity in both settings, suggesting that modulation of LAG-3 can potentiate anti–PD-1 efficacy (114). Several clinical trials targeting LAG-3 are currently ongoing. A randomized, double-blind phase II/III study (NCT05785767) is investigating fianlimab (αLAG-3) combined with cemiplimab (αPD-1) as first- line therapy for advanced NSCLC with PD-L1≥50%, including patients previously treated with PD-1/L1 or CTLA-4 inhibitors. Another randomized, double-blind phase II/III trial (NCT05800015) is comparing fianlimab plus cemiplimab combined with chemotherapy versus cemiplimab plus chemotherapy in first-line treatment of advanced NSCLC, also including pretreated populations. In addition, a bispecific antibody targeting PD-1 and LAG-3 (RO7247669) is currently being evaluated in a clinical trial (NCT04140500) for its preliminary antitumor activity in NSCLC patients who have previously received PD-1/L1 inhibitors. Collectively, these findings highlight LAG-3 as a promising target in overcoming primary and acquired resistance to PD-1/PD-L1 blockade, and support the development of dual LAG-3/PD-1 targeting strategies as a next-generation immunotherapeutic approach in NSCLC (**Table 1**).

### 4.2 αPD-1/L1 combined with other immunotherapies

Intratumoral administration of CCL21-modified DCs has been shown to promote CD8^+^T cells and Th1 cells infiltration into the tumor microenvironment of ICI-resistant NSCLC mouse models, while the combination of CCL21-DCs with αPD-1 therapy further enhances antitumor efficacy (115). A recent phase I clinical trial reported that monocyte-derived dendritic cells genetically engineered to express the chemokine CCL21, when combined with pembrolizumab, may overcome immunotherapy resistance; this trial is currently ongoing (116). In murine NSCLC models, intratumoral delivery of CXCL9/10-expressing DCs in combination with ICI can overcome resistance to ICI therapy and induce systemic tumor-specific immunity, primarily through CXCR3-mediated recruitment of intratumoral CD4^+^ and CD8^+^T cells (117). Identification of novel antigenic epitopes and construction of epitope-based DC vaccines also represent critical strategies for reversing αPD-1 resistance. In murine NSCLC, prediction of mutation-associated neoepitopes from ASB-XIV identified a mutated Phf3 peptide as an immunogenic epitope. Vaccination with mPhf3-loaded DCs combined with αPD-1 therapy significantly inhibited tumor growth in αPD-1–resistant mouse models (118).

In the ATALANTE-1 trial, a two-stage, open-label, randomized controlled study, a cancer vaccine significantly improved survival in HLA-A2–positive advanced NSCLC patients with secondary resistance (35). The NEO-PV-01 cancer vaccine, combined with chemotherapy and αPD-1, effectively induced neoantigen-reactive T cell activation with sustained cytotoxicity (119). Furthermore, in a single-arm, open-label phase I trial (NCT03215810) involving 20 advanced NSCLC patients who experienced disease progression after nivolumab monotherapy, the combination of tumor-infiltrating lymphocytes (TILs) with nivolumab achieved confirmed responses in 3 of 13 evaluable patients, tumor burden reduction in 11 patients, and complete responses in 2 patients—both maintaining remission beyond 1.5 years—indicating that TIL-based adoptive cell therapy combined with ICI may be a feasible strategy for overcoming αPD-1 resistance in NSCLC (120). Preclinical and clinical studies demonstrate that combining ICI with other immunotherapeutic strategies can effectively overcome primary and acquired resistance in NSCLC. These findings highlight the promising potential of rational ICI-based combination regimens to enhance antitumor immunity and improve outcomes for patients with advanced disease.

### 4.3 αPD-1/L1 combined with targeting the immunosuppressive microenvironment

#### 4.3.1 αPD-1/L1 combined with targeting myeloid cells

The TAM receptors (Tyro3, Axl, and MerTK) are members of the receptor tyrosine kinase family and can promote the polarization of TAMs toward the M2 phenotype, thereby mediating pro-tumorigenic effects (121). A phase II clinical study (NCT02954991) reported that in NSCLC patients who experienced disease progression after ICI therapy, treatment with sitravatinib plus nivolumab demonstrated promising clinical activity, with a median OS of 15 months (1-year and 2-year OS rates of 56% and 32%, respectively), a median PFS of 6 months, an ORR of 16% (11/68, including 2 complete responses), and a median duration of response (DOR) of 13 months (122). Building on these results, a phase III clinical trial (NCT03906071) evaluated the efficacy of sitravatinib combined with nivolumab in non-squamous NSCLC patients who progressed after platinum-based chemotherapy plus ICI. The findings indicated that sitravatinib plus nivolumab improved OS compared with docetaxel, although the difference was not statistically significant, suggesting the need for further preclinical studies to investigate resistance mechanisms (123). Additionally, the SAFFRON-301 phase III trial (NCT04921358) is assessing the efficacy of tislelizumab combined with sitravatinib versus docetaxel monotherapy in advanced/metastatic NSCLC patients who progressed after platinum-based chemotherapy plus ICI (124). Another ongoing phase III trial (NCT04471428) is evaluating the efficacy of atezolizumab in combination with cabozantinib compared with docetaxel monotherapy (125). Meanwhile, some other related clinical trials are also underway (**Table 2**).

**Table 2:** Clinical trials of αPD-1/L1 combined with targeting of immunosuppressive populations in ICI-resistant NSCLC.

NCT identifier	Eligible patient	Treatment strategy	Phase	Status	Result
Target myleoid cells					
TAMR					
NCT02954991	Prior treatment with ICI therapy	Sitravatinib & Nivolumab	II	Terminated	Good safety profile, clinical antitumor activity
NCT03906071	NSCLC progressing on prior αPD-1/L1 therapy, and tumor confirmed negative for EGFR, ROS1, and ALK alterations or fusions	Sitravatinib & Nivolumab	III	Active, not recruiting	Primary endpoint (INV-PFS) not achieved
NCT04921358	NSCLC progressing on prior αPD-1/L1 therapy	Tislelizumab& Sitravatinib	III	Terminated	Not yet announced
NCT04471428	Metastatic NSCLC progressing on prior αPD-1/L1 therapy	Atezolizumab& Cabozantinib	III	Completed	No OS improvement
NCT04681131	NSCLC progressing on prior αPD-1/L1, EGFR, or ALK Inhibitor therapy	CAB-AXL-ADC&αPD-1	II	Completed	\
CD47					
NCT04881045	Advanced or metastatic NSCLC progressing on prior αPD-1/L1 therapy	PF-07257876	I	Completed	Well tolerated, manageable toxicity, and modest antitumor activity
CD73					
NCT05431270	NSCLC progressing on prior αPD-1/L1 therapy	Mavrostobart& Tislelizumab	I/II	Recruiting	\
NCT06984588	Locally advanced or metastatic NSCLC without receiving any treatment	Uliledlimab& Toripalimab	II/III	Recruiting	\
Cytokines					
VEGF					
NCT04471428	NSCLC progressing on prior αPD-1/L1 therapy	Atezolizumab&Cabozantinib	III	Coxmpleted	No significant improvement in OS
NCT04340882	NSCLC progressing on prior αPD-1/L1 therapy	Pembrolizumab& Ramucirumab& Docetaxel	II	Active, not recruiting	Well tolerated
NCT03689855	NSCLC progressing on prior ICI therapy	Ramucirumab&Atezolizumab	II	Completed	Not yet announced
NCT05633602	NSCLC progressing on prior αPD-1/L1 therapy	Ramucirumab &pembrolizumab	III	Active, not recruiting	\
NCT04736823	Advanced NSCLC progressing on prior αPD-1/L1 therapy	AK112	II	Recruiting	Good anti-tumor activity and safety
NCT06616584	Recurrent NSCLC receiving αPD-1/L1 therapy	Ramucirumab& Cemiplimab	II/III	Recruiting	\
Others					
NCT04725474	Advanced or recurrent NSCLC progressing on prior αPD-1/L1 therapy	Visugromab&Nivolumab	I/II	Active, not recruiting	Durable and deep responses in some patients

This table lists only the characteristics of the eligible patients related to ICI resistance. Other characteristics of the eligible patients in the cohort can be found on ClinicalTrials.gov.

#### 4.3.2 αPD-1/L1 combined with targeting Tregs

Tregs are key mediators of ICI resistance within the TME. In PD-L1–resistant mouse models, αPD-L1 treatment preferentially activates and expands Tregs, whereas systemic depletion of Tregs can restore the therapeutic efficacy of αPD-L1 (126). Although dual blockade of the PD-1 and CTLA-4 immune checkpoints can effectively suppress Treg function and activate CD8^+^T cells, it is associated with considerable adverse effects. Recent studies have demonstrated that therapeutic depletion of the CCR8^+^Treg subset combined with αPD-1 can efficiently activate dendritic cells and enhance CD8^+^T cells cytotoxicity, yielding pronounced antitumor effects in various NSCLC mouse models (127). Moreover, multiple studies confirm that highly immunosuppressive Treg subsets play a critical role in mediating ICI resistance in NSCLC patients, suggesting that targeting these Treg subsets in combination with αPD-1/PD-L1 therapy may provide a promising strategy to overcome immune resistance.

#### 4.3.3 αPD-1/L1 combined with targeting CAFs

Excessive IFN-γ stimulation induces the expansion of apCAFs within the tumor microenvironment, which recruit FOXP1^+^ Tregs via the PD-L2–RGMB axis, thereby contributing to αPD-1 resistance in NSCLC patients. Targeting RGMB and reprogramming apCAFs can reverse apCAF-mediated immunotherapy resistance and exert synergistic antitumor effects (64). Furthermore, multiple studies have confirmed that various CAF subsets, including POSTN^+^ CAFs, COL11A1^+^ CAFs, FAP^+^αSMA^+^ CAFs, and MYH11^+^αSMA^+^ CAFs, play critical roles in mediating ICI resistance in NSCLC patients (29, 53, 128). Therefore, the development of therapies targeting specific CAF subsets in combination with ICI holds promise for effectively overcoming immunotherapy resistance.

#### 4.3.4 αPD-1/L1 combined with targeting cytokines

In *EGFR*-mutant NSCLC, TGF-β signaling is upregulated via EGFR activation and subsequent ERK1/2–p90RSK phosphorylation. TGF-β directly suppresses CD8^+^ T cell infiltration, proliferation, and cytotoxicity both *in vitro* and *in vivo*, whereas combined blockade using anti–TGF-β and αPD-1 antibodies markedly enhances the antitumor function of CD8^+^T cells (129). In *PTEN*-deficient NSCLC, αPD-1 resistance can be effectively overcome by combining poly(I:C) + R848 with αTGF-β, which inhibits tumor growth and potentially converts αPD-1–resistant tumors into αPD-1–responsive tumors (94). An ongoing clinical study (NCT04725474) demonstrated that the GDF-15 neutralizing antibody visugromab combined with nivolumab can overcome immunotherapy resistance in NSCLC patients, enhancing IFN-γ signaling within the tumor microenvironment and promoting GZMB^+^ CD8^+^ T cell infiltration (130).

Targeting the VEGF signaling pathway in combination with ICI has been shown to enhance antitumor efficacy in lung cancer (131). However, the therapeutic potential of such combinations in ICI-resistant NSCLC remains under active investigation. A phase II study (NCT02501096) evaluating pembrolizumab plus lenvatinib demonstrated promising antitumor activity in advanced NSCLC (132). Nevertheless, this efficacy was not confirmed in the phase III LEAP-008 trial (NCT03976375), where the same combination failed to achieve a meaningful survival benefit in patients who had progressed after prior PD-1/L1 therapy (133). Similarly, the phase III CONTACT-01 trial (NCT04471428) assessing atezolizumab plus cabozantinib in previously treated NSCLC patients also reported no significant improvement in OS (134). In contrast, the phase II Lung-MAP S1800A trial (NCT03971474) showed that pembrolizumab combined with the VEGFR2 antagonist ramucirumab significantly improved OS compared with standard therapies in patients who had progressed following checkpoint inhibitor treatment (135). The ongoing phase III Pragmatica-Lung trial (NCT05633602) aims to further validate this combination in a larger cohort. Additionally, a multicenter phase II trial (NCT04736823) evaluated AK112, a novel PD-1/VEGF bispecific antibody, in combination with platinum-based chemotherapy in patients with advanced NSCLC who had progressed on prior PD-1/L1 inhibitors. The results demonstrated encouraging antitumor efficacy and acceptable safety, particularly in those with acquired resistance to previous ICI therapy (136). Collectively, these studies indicate that while VEGF-targeted combinations may not universally overcome ICI resistance, dual targeting of PD-1 and angiogenic pathways—especially through bispecific antibodies—represents a promising direction for future therapeutic development in resistant NSCLC.

### 4.4 αPD-1/L1 combined with chemotherapy

Multiple clinical trials have demonstrated that ICI, including pembrolizumab, atezolizumab, and cemiplimab, in combination with chemotherapy can effectively prolong median OS in NSCLC patients (137–140). Preclinical studies also support the role of ICI-chemotherapy combinations in overcoming immune tolerance in NSCLC. Srivastava et al. demonstrated that oxaliplatin can activate macrophages to express T cells–recruiting chemokines, promote ROR1-CAR T cells infiltration into the tumor core, and restore tumor sensitivity to αPD-L1 therapy, resulting in durable antitumor effects (141). Similarly, paclitaxel combined with αPD-1 can reverse *KRAS* ^G12D^-mediated αPD-1 resistance and enhance antitumor activity (78). However, in *Kras* ^G12C^ NSCLC mouse models, the combination of αPD-L1 and docetaxel did not demonstrate enhanced antitumor effects (142).

Clinically, a multicenter, open-label, randomized phase III trial (NCT03088540) demonstrated that in patients with PD-L1 ≥ 50%, cemiplimab monotherapy achieved a median OS of 26.1 months versus 13.3 months with chemotherapy (HR 0.57, 95% CI 0.46–0.71; *P* < 0.0001). Notably, for patients who experienced disease progression after first-line cemiplimab, cemiplimab combined with chemotherapy has been suggested as a potential second-line therapeutic option (143). Although there are relatively few studies specifically evaluating the use of ICI combined with chemotherapy in ICI-resistant NSCLC, this is mainly because chemotherapy is often used together with other combination therapy regimens as the first-line treatment for NSCLC patients. Overall, these findings highlight the important role of ICI–chemotherapy combinations in overcoming resistance and extending survival in NSCLC. Future research should focus on personalized treatment approaches, optimizing combination timing, and identifying biomarkers that predict which patients are most likely to benefit from this strategy.

### 4.5 αPD-1/L1 combined with nanotechnology

Limited drug penetration is also a critical contributor to αPD-1 immunotherapy resistance. Advances in nanotechnology have offered promising strategies to overcome this challenge. Yen and colleagues developed a novel gelatinase-responsive nanoparticle system that co-delivers αPD-1 and a TGF-β receptor inhibitor directly to the tumor site, thereby enhancing tumor-specific cytotoxicity (144). Another innovative nanomedicine, SGT-53—a plasmid DNA nanocomplex carrying wild-type human TP53—has been shown to augment the antitumor efficacy of PD-1 blockade in TP53-matched, PD-1-resistant lung cancer mouse models. SGT-53 restores normal p53 function, reduces immunosuppressive M2 macrophages, and thereby reinstates antitumor immune responses against lung cancer cells. The combination of SGT-53 with αPD-1 therapy holds potential to improve response rates in ICI-resistant lung cancer patients (145). Additionally, nanoparticle-mediated radiotherapy using NBTXR3 combined with αPD-1, αTIGIT, and αLAG-3 triple blockade has been demonstrated to effectively reverse αPD-1 resistance in lung cancer models (16).

## 5 Safety and toxicity of combination immunotherapy

Dual immune checkpoint blockade can synergistically enhance T-cell activation and augment antitumor immunity by concurrently releasing distinct inhibitory pathways. However, compared with monotherapy, such combinations are associated with a substantially higher incidence of immune-related adverse events (irAEs) and a broader spectrum of organ involvement. Clinical data indicate that nivolumab plus ipilimumab results in an increased frequency of grade ≥3 irAEs, including elevated lipase, colitis, adrenal insufficiency, and pneumonitis, reflecting the intensified immune activation caused by dual blockade. Nevertheless, most toxicities are reversible and manageable with early recognition and appropriate intervention (102, 103). In the S1400F trial, durvalumab plus tremelimumab in previously treated advanced squamous NSCLC was associated with a 34% incidence of grade ≥3 treatment-related adverse events (TRAEs), primarily hematologic and pulmonary toxicities, including two deaths from immune-mediated pneumonitis, underscoring the need for vigilant monitoring of lung-related toxicity (104). Emerging bispecific antibodies, such as cadonilimab (PD-1/CTLA-4 bispecific), have demonstrated improved overall tolerability in early-phase studies. Their toxicity profiles are consistent with those of dual checkpoint inhibitors but with a lower incidence of severe AEs (<12%), suggesting that antibody engineering and optimization of affinity or dosing may mitigate toxicity without compromising efficacy (105). Beyond CTLA-4, novel checkpoint inhibitors targeting TIGIT, TIM-3, and LAG-3 have shown manageable and reversible toxicities comparable to αPD-1 monotherapy in early trials, though larger and longer-term studies are warranted to define the incidence and features of rare or delayed irAEs.

ICI-based combination immunotherapies—including therapeutic cancer vaccines, personalized neoantigen vaccines, and adoptive T-cell transfer—have demonstrated promising potential in overcoming resistance with generally superior tolerability compared to small-molecule or dual-ICI regimens. In the ATALANTE-1 trial, the HLA-A2–restricted multi-epitope vaccine OSE2101 exhibited good tolerability in chemotherapy- or ICI-refractory advanced NSCLC, with a significantly lower rate of grade ≥3 TRAEs than chemotherapy (11.4% vs. 35.1%), most commonly mild fever and no treatment-related deaths (35). The personalized neoantigen vaccine NEO-PV-01, in combination with pembrolizumab and chemotherapy, also showed a favorable safety profile, with mainly gastrointestinal and hematologic toxicities and no frequent occurrences of severe irAEs such as colitis or pneumonitis (119). In a phase I study (NCT03215810) of TILs plus nivolumab, most grade ≥3 toxicities were attributed to preconditioning radiochemotherapy rather than T-cell infusion itself, and most events resolved within one-month post-infusion, suggesting a relative safety advantage of cell therapy based ICI combinations (120).

Combining ICIs with molecule targeted inhibitors (e.g., MEK, VEGFR, or multi-target tyrosine kinase inhibitors) can enhance efficacy but often results in increased treatment-related toxicity. For instance, in the MRTX-500 trial, 91% of patients experienced TRAEs and 60% developed grade 3–4 toxicities, including hypertension and diarrhea (122). Similar findings have been reported in other phase III and early-phase trials, where a substantial proportion of patients discontinued treatment due to adverse events or experienced serious toxicities, and in some cases, trials were terminated early due to an unfavorable safety-to-benefit ratio (124). Common toxicities associated with such regimens include hypertension, diarrhea/colitis, hepatic dysfunction, hematologic toxicity, and pneumonitis, which may be alleviated through dose optimization, sequential administration, or extended dosing intervals.

Combinations targeting PD-1/PD-L1 and VEGF/VEGFR pathways—which aim to reprogram tumor vasculature and remodel the immune microenvironment—have shown synergistic efficacy but are often accompanied by substantial vascular-related toxicities. In LEAP-008(NCT03976375), the combination of lenvatinib plus pembrolizumab was associated with a 91.7% TRAE rate, with grade 3–4 events in 55.8% of patients and several grade 5 fatal events (e.g., pulmonary embolism, cardiac arrest), and a higher treatment discontinuation rate than chemotherapy (133). In CONTACT-01(NCT04471428), atezolizumab plus cabozantinib resulted in grade 3–4 TRAEs in 39.5% of patients and a 2.2% treatment-related mortality rate (134). By contrast, the IMpower150 study of atezolizumab plus bevacizumab did not reveal any unexpected safety signals, with irAE incidence comparable to PD-L1 monotherapy (12). The next-generation bispecific molecule AK112 demonstrated improved tolerability in early-phase studies, with grade ≥3 TRAEs in only 26.5% of patients and a 2.4% discontinuation rate—significantly lower than those observed with traditional dual-ICI regimens (grade ≥3: 47% and 52%; discontinuation: 15% and 19%)—highlighting the potential of molecular design and target selection to refine safety profiles (136, 146, 147).

Overall, although diverse combination immunotherapy strategies hold clear potential for overcoming resistance in NSCLC, the type and severity of adverse events are closely linked to the degree of immune activation and the inherent toxicity of the combined agents. Dual ICI and ICI–VEGF/TKI combinations often result in greater systemic immune and vascular/metabolic toxicity, whereas vaccine- or T cell–based approaches generally exhibit better tolerability. Advances in bispecific antibody engineering and next-generation immune modulators may further improve safety without compromising efficacy. Looking forward, standardized AE assessment, biomarker-driven patient stratification, optimized dosing and scheduling, and enhanced long-term and real-world pharmacovigilance will be essential to fully realize the therapeutic potential of combination immunotherapy in overcoming resistance in NSCLC.

## 6 Conclusion

ICI have brought groundbreaking advances to the treatment of NSCLC by reactivating the host immune system and inducing sustained antitumor immune responses, significantly improving survival outcomes in a subset of patients. However, the clinical application of ICI monotherapy remains limited due to the widespread occurrence of immune resistance. Primary resistance often arises from the innate inability of certain patients to respond to ICI, a phenomenon closely associated with the heterogeneity of the TME. Therefore, identifying patients suitable for ICI and establishing reliable predictive biomarkers are critical in clinical practice. Currently, PD-L1 expression is one of the most commonly used biomarkers to guide ICI therapy, although its predictive value remains controversial, as some PD-L1–negative patients still derive clinical benefit. Recently, tumor mutational burden has emerged as a potentially more predictive biomarker, reflecting, to some extent, tumor antigen load and immunogenicity. Advances in high-throughput sequencing have also identified specific gene mutations, such as *STK11*, *KEAP1*, and *KRAS*, which are closely associated with ICI efficacy, providing new avenues for precision immunotherapy. In contrast, acquired resistance presents greater challenges, typically emerging after an initial clinical response to ICI. Its mechanisms are complex, involving dynamic adaptations of the TME, remodeling of immune cell composition and function, tumor clonal evolution, and immune escape. A thorough understanding of the mechanisms driving acquired resistance and identification of its key contributors are crucial for developing more effective combination strategies to delay or reverse immune resistance.

While the resistance encountered with ICI monotherapy is discouraging, it is, to some extent, understandable. Cancer is a highly heterogeneous and complex disease, and achieving clinical cure through a single treatment is inherently challenging. Consequently, combination strategies are widely recognized as a critical approach to overcoming ICI resistance. Multiple combination modalities are actively being explored, including ICIs combined with other immunotherapies such as CAR-T, TCR-T, cancer vaccines, and TILs, as well as combinations with conventional chemotherapy, radiotherapy, or targeted therapies. These approaches aim to intervene synergistically from multiple angles to overcome key limitations imposed by immune resistance. In summary, a deep understanding of the mechanisms underlying ICI resistance provides a solid foundation for designing more rational and effective treatment strategies. With ongoing advances in tumor immunology and molecular biology, personalized and precision combination therapies are expected to enable more NSCLC patients to derive meaningful benefit from immunotherapy.
